# Acknowledgment to the Reviewers of *Eur. Burn J.* in 2022

**DOI:** 10.3390/ebj4010003

**Published:** 2023-01-13

**Authors:** Eur. Burn J. Editorial Office

**Affiliations:** MDPI AG, St. Alban-Anlage 66, 4052 Basel, Switzerland

High-quality academic publishing is built on rigorous peer review. *Eur. Burn J.* was able to uphold its high standards for published papers due to the outstanding efforts of our reviewers. Thanks to the efforts of our reviewers in 2022, the median time to first decision was 26 days and the median time to publication was 53 days. Regardless of whether the articles they examined were ultimately published, the editors would like to express their appreciation and thank the following reviewers for the time and dedication that they have shown *Eur. Burn J.*:
Adam ReichLuigi SchiraldiAgnieszka KulikM. K. NieuwenhuisAgnieszka LasotaMaheswari MuruganandamAgnieszka Owczarczyk-SaczonekMahsa BagheriAlberto BollettaMamta ShahAlessandro FailoMarco ContardiAlette E. E. De JongMarjorie FlahautAlina ZurekMarwa ElgendyAlperen S. BingoelMary Beth Browning MonroeAndrew HollandMathers JonathanAndrzej HeckerMaurizio StellaAndrzej KobylińskiMichael FroschAnna BocchinoMichael GiretzlehnerAthina LavrentievaMichael SticherlingBarclay T. StewartMichele AltomareCecilia Li-TsangMihaela PerteaChristian SmolleMin YaoChristine J. AndrewsMomcilo JankovicChristoph WallnerMostafa Amini RaraniClemens SchiestlNadia DepetrisColleen M. RyanNancy Van LoeyDandan GuoNathalie AndréDaniel M. SchneiderNicco KrezdornDavid MackieOliver ThammDiana HarcourtPablo A. Cantero-GarlitoEdoardo RaposioPeter M. VogtEduardo GusPhilip EffraimEdward RenRachel KornhaberEvan RossRamon SirventFelicia N. WilliamsRandolph StoneFlorian BucherRichard David HaywardFredrik HussRichard Wong-SheGuillermo LaheraRolf LeferingHans-Oliver RennekampffSarah McGarryHon-Yi ShiSatish ToteyIngrid SteinvallSeon-Cheol ParkIrene Carrillo MurciaSerge JennesJeremy GovermanShankar Jaikishan EvaniJihye LeeShin-Chen PanJohn FisherShiva AkbarzadehJohn T. SchulzSteve JefferyJonathan BayuoSujata MohantyJose-Antonio Mirón-CaneloThomas BirngruberJosef HaikYangtian YiJuan Pedro Martínez RamónYaron ShohamJulie CaffreyYaw Duah BoakyeJun WuYong-Ming YuJyrki VuolaYuji TaniKaitlin A. PruskowskiYuko OnoKarel ClaesYvonne SingerKarol KonaszewskiYvonne WilsonKevin K. ChungZephanie F. TyackLars-Peter KamolzZephanie TyackLing MaZhe LiLuca Simione



